# In Nature's School

**Published:** 1889-07-20

**Authors:** 


### The Doctor's Pulpit.

### IN NATURE'S SCHOOL.

### "First the Blade."

There is not a schoolboy in the remotest village in Britain who does not understand by practical experience that "the blade" of the growing corn is first to show itself above ground, and that the "full corn in the ear' can only be reaped several months later. Minerva may have sprung full-fledged from the head of Jove; but that is by no means a phenomenon of every day occurrence. A "waiting game" is what the majority of people have to learn to play, and have to practice pretty constantly. Inexperience and impatience are youthful companions whose fingers are always itching to be at the flowers and fruits of life. Sober knowledge understands that the ground must first be prepared and the seed sown; and that then there must be months or years of expectation and hope before the day of profit and reward dawns.

If men could have chosen the lines of their own destiny, there can be little doubt that they would have dispensed with both the seed-sowing and the waiting, and would have had everything always ready for immediate use and pleasure. With different beings from men, it is conceivable that such an arrangement might have worked very well; but being what we are, it is certain that both health and happiness would have been ruined for us if we could have had everything we wanted without work, and merely for the asking. Philosophers have gravely discussed whether the pursuit of an object or the gaining of it is the real essence of life. Many of them have decided that the pleasure is in the pursuit largely or entirely, and not in the gained object. The present writer does not agree with that. He holds that not mere pursuit, but successful pursuit brings the highest satisfaction. To be overloaded with everything that heart could wish without effort is perhaps one of the most unhappy, as it certainly is one of the most injurious, conditions a man or woman can be placed in. But to have worthy objects of pursuit, to pursue them honourably and honestly, and with all the heart and energy of which a man is capable, and after due and strenuous effort to succeed in the highest degree; that is the kind of life which is really worth living. Of course it is not affirmed that failure makes life not worth living. Repeated checks and failures may be the needed discipline which prepare man or woman for the noblest success.

There is no department of life or knowledge in which a man must not begin at the beginning. He himself was an infant before he was a boy, and a boy before he became a man. He began his pursuit of knowledge by learning A, B, C; and as each new branch of study was entered upon, the elements were always the first to be learnt. The numerals in arithmetic, the declensions in grammar, the simplest facts in science—that was the order of it—"First the blade." If a man would know what manner of man he is in physical and moral constitution, he must begin at the beginning. This is especially true if he aspires to a knowledge of his physical constitution, as every rational man does aspire. For, however much the mind may be the real thinking, planning, and actual essence of the man, the physical organism is at least the indispensable instrument by means of which the mind works, at any rate in the earthly sphere. The man, therefore, who pays no heed to his body is exactly on a par with the engineer who knows nothing of his engine, or the carpenter who never looks to the condition of his tools.

There are two kinds of knowledge which a man should have of his own constitution. Most people speak of these as "scientific" and "practical," but that is an illogical distinction which is neither scientific nor practical. All practical knowledge, so far as it is knowledge, is truly scientific, the most scientific, in fact, of all knowledge. It is a question, indeed, how far any kind of knowledge is worth calling scientific which has not been put to the proof of practice. The real distinction between these two knowledges is between "academical" and "practical." A man may know things academically, who is at the same time a very poor creature in practice. Now, it is hardly possible for any person, other than a medical man, to know much of his physical organism academically. The structure and functions of the human body are so various, so complicated, and so difficult to master, that not one layman in ten thousand has either the opportunity or the disposition to study them with any degree of thoroughness. But the practical knowledge of a man's own constitution is a very different thing. Such knowledge he has the most ample opportunities for acquiring, and that in very full measure. Does he not carry his own body about with him everywhere? Does he not know all that he eats and the effect it has upon him? Is he not familiar with his own daily drink, and with the consequences it produces? Does he not know his own capacity for walking, running, leaping, cricket-playing, wood-chopping, and every other form of physical exercise? Does he not know whether he is a good or a bad sleeper, and what things promote and what prevent sleep? Has he not again and again proved his own capacity, or want of capacity, for hard study, steady work at the desk, or severe and prolonged worry? Surely, in these days of intelligent and strenuous thinking, a man must be a veritable Rip Van Winkle, fast asleep year in and year out, if he has not acquired some rational knowledge of himself of this practical kind. It may be that with many such knowledge is very limited, purely in the "blade" stage, and that it is under-valued on that account. But it should by no means be despised, however limited it be, provided it is reasonably accurate. "A little knowledge" is better than none at all. Even if to foolish creatures a "little knowledge" may sometimes be a dangerous thing, yet to the world at large it never can be half so dangerous as total ignorance. A man with one eye may not see so clearly as a man with two; but undoubtedly he sees much better than one who has no eyes at all, either good or bad.

From the earliest ages philosophers and moralists have insisted upon the prime necessity of self-knowledge in the mental sphere. Will any physiologist or medical man have the hardihood to declare that what is the first essential in the sphere of mind is either useless or dangerous in the sphere of bodily activity? Anyone who should so declare would thereby place himself completely outside the range of men whose opinions are worth even so much as debating. When Bishop Berkeley had by rigorous logic brought himself to the conclusion that there was no such thing as matter, the argument against him was wittily summed up and closed in a single sentence: "If the bishop says there is no matter, it does not matter what the bishop says." And so if any man affirms that a little knowledge is dangerous, it may be highly dangerous to have a little knowledge of him. People who have private closets that are always kept tightly locked afford reasonable ground for suspicion that, in ultimate analysis, they belong to the Bluebeard class.

The blade of corn or grass has within it the "promise and potency" of the full corn in the ear. So is it with every man who has begun to take the trouble to acquire a rational and practical knowledge of himself. He may in time, if he uses all available helps, both academical and experiential, come to understand his own physical organism with great intelligence. He may thus not only save himself from an infinity of worrying anxieties, but also promote his own efficiency in business, and at the same time both preserve his health and prolong his life. Just as a judicious rider, who is familiar with the temper and other qualities of his horse can get the most work out of him with the least wear and tear, so will the man who thoroughly understands his own organism bring himself to the highest level of efficiency, and maintain himself there for the longest period. Knowledge is not only the "parent of eloquence," as Beaconsfield said, but it is also the source and origin of a hundred other kinds of excellence. He who knows can often do: He who knows not can never do except by chance and accident.

